# The Relationship among Carotid Artery Remodeling, Cardiac Geometry, and Serum N-Terminal Pro–B-Type Natriuretic Peptide Level in Asymptomatic Asians: Sex-Differences and Longitudinal GEE Study

**DOI:** 10.1371/journal.pone.0131440

**Published:** 2015-07-01

**Authors:** Chen-Yen Chien, Chuan-Chuan Liu, Helen L. Po, Chih-Hsuan Yen, Charles Jia-Yin Hou, Jen-Yuan Kuo, Chung-Lieh Hung, Shoei-Shen Wang, Hung-I Yeh, Carolyn S. P. Lam

**Affiliations:** 1 Division of Cardiovascular Surgery, Mackay Memorial Hospital, Taipei, Taiwan; 2 Mackay Medical College, New Taipei City, Taiwan; 3 Health Evaluation Center, Mackay Memorial Hospital, Taipei, Taiwan; 4 Department of Medical Technology, Yuanpei University of Science and Technology, Hsin-Chu, Taiwan; 5 Graduate Institute of Health Care Organization Administration, College of Public Health, National Taiwan University, Taipei, Taiwan; 6 Department of Neurology, Mackay Memorial Hospital, Taipei, Taiwan; 7 Cardiovascular Division, Department of Internal Medicine, Mackay Memorial Hospital, Taipei, Taiwan; 8 Mackay Medicine, Nursing and Management College, Taipei, Taiwan; 9 Department of Health Industry Management, Kainan University, Taoyuan, Taiwan; 10 Institute of Clinical Medicine, National Yang-Ming University, Taipei, Taiwan; 11 Department of Surgery, National Taiwan University Hospital, Taipei, Taiwan; 12 Yong Loo Lin School of Medicine, NUS, National University Health System, Singapore, Singapore; National Cardiovascular Center Hospital, JAPAN

## Abstract

**Background:**

Carotid artery remodeling is known to be associated with a variety of cardiovascular diseases. However, there is limited information regarding gender differences in carotid remodeling. We sought to investigate the associations among blood pressure (BP), carotid artery remodeling and cardiac geometries, and further explore gender differences.

**Materials and Methods:**

In a large cohort of asymptomatic adults undergoing routine health screening with repeated observations, we related measures of carotid artery diameter (CCAD) to various BP components, cardiac geometries and blood N-terminal pro-brain natriuretic peptide (NT-proBNP) level, both from baseline cross-sectional and longitudinal dataset using generalized estimating equations (GEE).

**Results:**

A total of 2,914 person-visits (baseline: n=998, mean age: 47 ± 8.9 years, 34% female) were studied (median: 6 ± 1.73 years follow up). We observed that CCAD was larger in men (p<0.01) and positively related to baseline age or all blood pressure components (including systolic BP [SBP], diastolic BP [DBP] and pulse pressure [PP], all p<0.01) even after accounting for clinical covariates, which did not change significantly at follow up (repeat-visit longitudinal GEE models). At baseline, per each increased unit of CCAD was associated with elevated LV mass index (β-coef: 6.72, with odds ratio [OR]: 1.47, 95% CI: 1.06 to 2.07 for ventricular hypertrophy; AUROC: 0.65, CCAD cut-off: 7.25mm) and NT-proBNP (β-coef: 5.35, OR: 4.22, 95% CI: 1.42 to 12.6 for >=300pg/mL; AUROC: 0.79, CCAD cut-off: 7.95mm, all p<0.05), which remained significant in multi-variate and longitudinal models. There was a prominent sex interaction (p for interaction with age and systolic BP: 0.004 and 0.028 respectively), where the longitudinal associations of age and systolic BP with increasing CCAD as more pronounced in women than men.

**Conclusion:**

These data demonstrated that carotid artery remodeling may parallel subclinical biomarker of cardiac dysfunction, and further showed greater effects of aging and higher blood pressure on such remodeling process in women than men. Further study is warranted to understand how this predisposition of elderly hypertensive women to vascular remodeling may play a role in clinical settings.

## Introduction

Age-related arteriosclerosis of the carotid arteries and chronically elevated arterial wall stress may result in vascular luminal expansion and degeneration [1–2] in terms of carotid artery remodeling. Indeed, cross-sectional studies have shown that increased carotid arterial stiffness is associated with advanced age, atherosclerosis, type II diabetes, metabolic syndrome, and hypertension [3–7]. Our previous cross-sectional study additionally demonstrated that carotid artery remodeling is associated with altered left ventricular geometry, higher serum biomarkers (brain natriuretic peptide) and the development of preserved ejection fraction heart failure [8]. However, data are limited regarding the longitudinal changes of carotid arterial diameter and associated biomarker changes with aging. In addition, the clinical determinants and sex differences in such artery remodeling process remain scarce so far. Prior studies have demonstrated sex differences in aortic root remodeling [9], but whether this also relates to carotid arterial remodeling is unclear. Carotid arteries are easily accessible for assessment by ultrasonography, and carotid ultrasound is increasingly used as a clinical tool for cardiovascular risk stratification. A better understanding of any sex differences, as well as the effects of age and key clinical determinants of carotid arterial remodeling, would therefore be useful.

Accordingly, we aim to investigate two major issues in this current study. The first, we aim to explore the association between carotid artery remodeling process and N-terminal pro-brain natriuretic peptide (Nt-proBNP), a subclinical surrogate for cardiac dysfunction, in an asymptomatic population. The second, we aim to examine the effect of sex on the associations among carotid artery diameter (CCAD), age, various blood pressure (BP) components (systolic BP [SBP], diastolic BP [DBP], pulse pressure [PP]), and clinical covariates in a cross-sectional and longitudinal fashion. We hypothesized that aging and arterial hypertension, both key cardiovascular risk factors associated with cardiac and central aortic remodeling, would be related to carotid arterial dilatation and paralleled elevation of Nt-proBNP, which may have sex differences in individuals at risk.

## Material and Methods

### Study population

**Fig 1:**
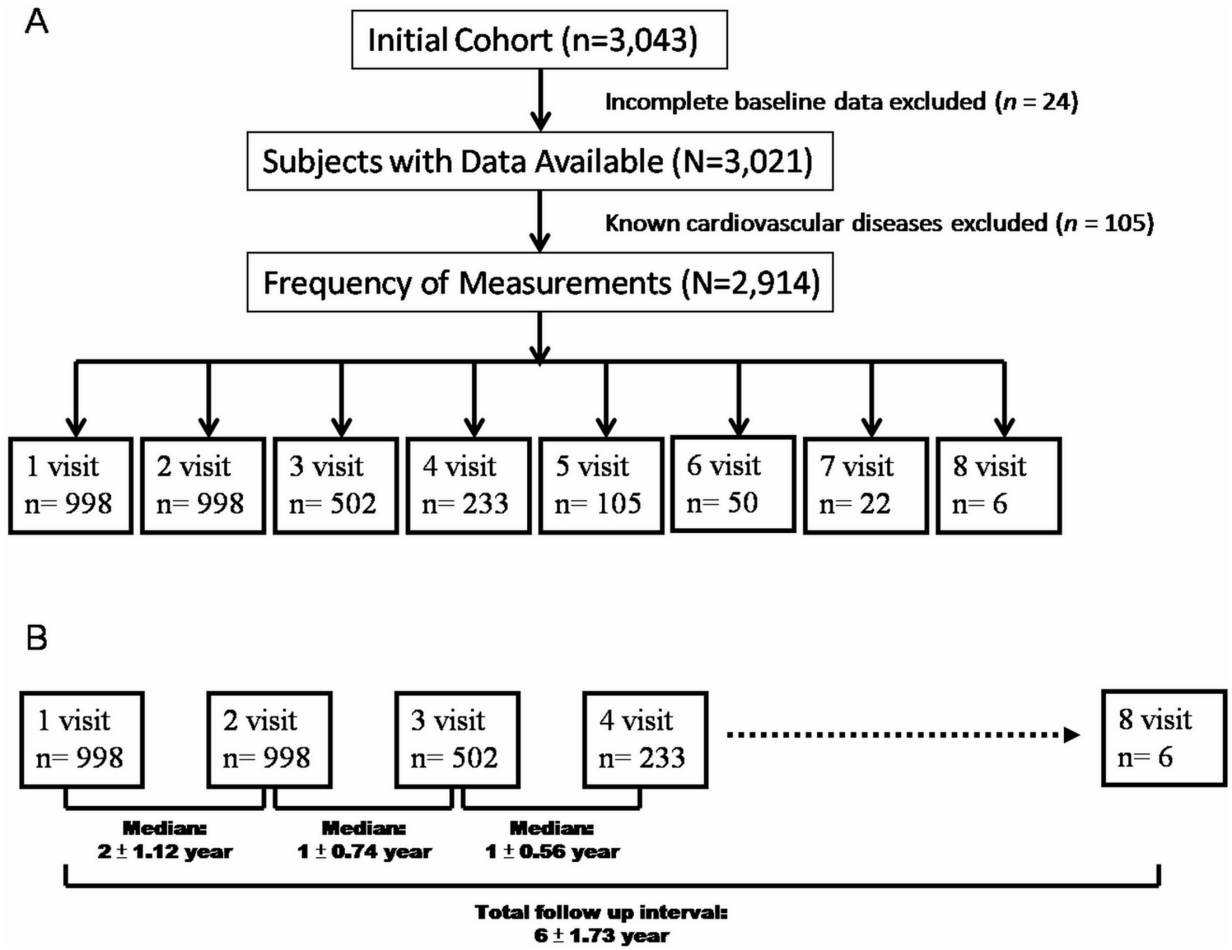
The flow-chart and time tables for baseline and longitudinal study participants enrollment.

**Fig 2:**
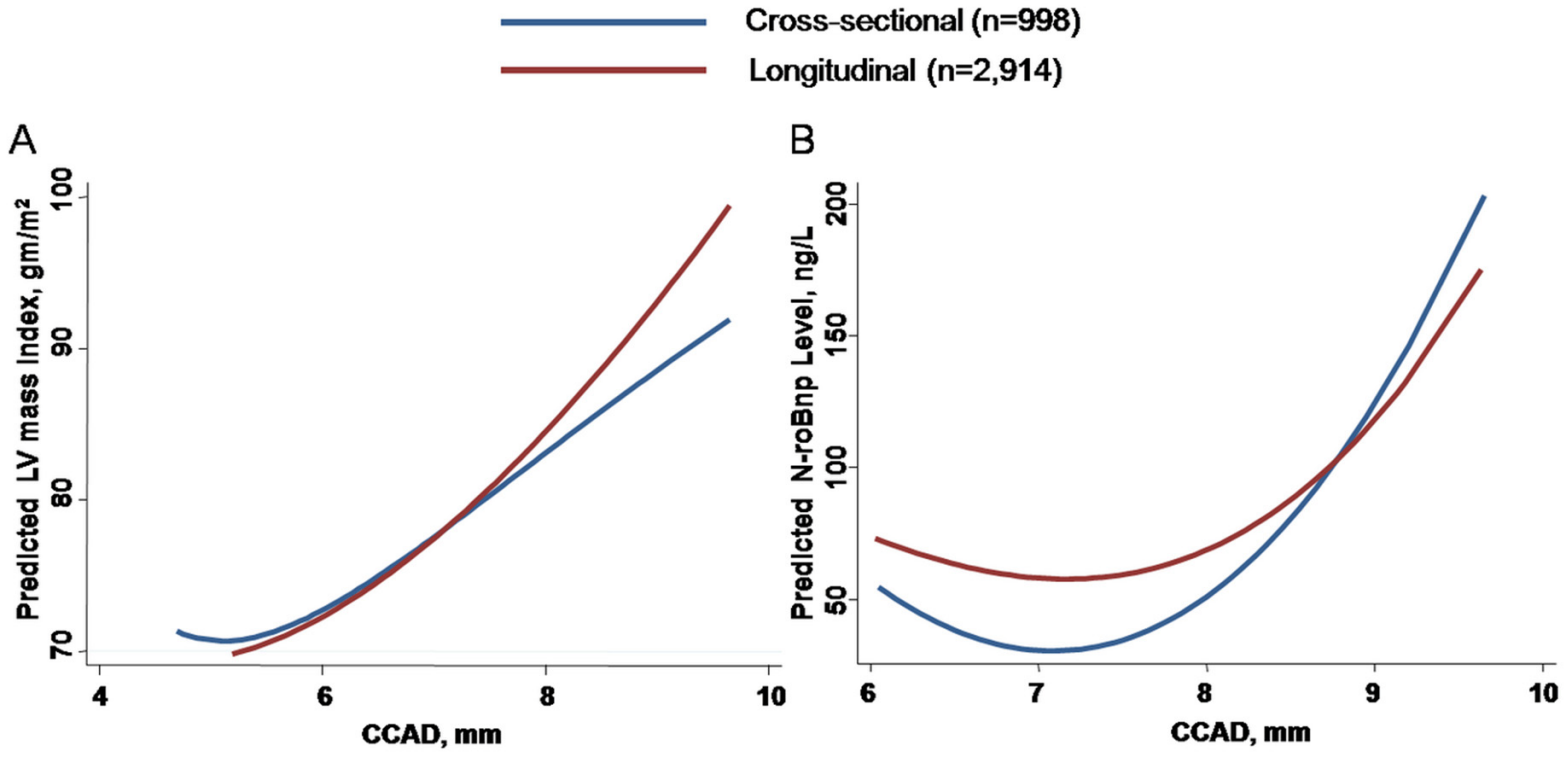
The fitting curves between baseline and serial longitudinal data of CCAD, LV mass index (A) and serum Nt-ProBNP level (B). Both showed a positive relationship with and without adjusting for confounders.

Consecutive participants who underwent annual cardiovascular health survey for primary preventive disease screening at a tertiary center (Mackay Memorial Hospital) in Northern Taiwan from January 2005 to January 2010 were eligible for this study (*n* = 3,014 eligible person-visits in total, Fig 1). We excluded participants with missing clinical or echocardiographic data (*n* = 24), and known or symptomatic cardiovascular disease (previous coronary artery disease, heart failure, atrial fibrillation or stroke; ongoing angina, palpitations or exercise intolerance) (*n* = 105). The remaining 2,914 asymptomatic person-visits (in 998 participants undergoing 2–8 sequential visits over a median of 6 years) constituted the final study sample (Fig 2).

During each visit, participants underwent detailed medical review, anthropometric measurements, blood sampling, echocardiography and carotid artery imaging. The presence of hypertension or diabetes was defined by either a confirmed history of the diagnosis or current regular medication usage. Regular exercise was defined as moderate exercise of at least 30 minutes, 3 times a week. This study was mainly retrospective in study design, all personal information was de-identified prior to data analysis and had been approved by the local ethics committee in accordance with the Declaration of Helsinki.

### Anthropometric measurements and determination of various blood pressure components

Height and weight were obtained routinely on the day of health check-up. Body mass index (BMI) and body surface area (BSA) were calculated as: BMI = weight/height^2^; BSA (m^2^) = 0.20247×height (m)^0.7256^×weight (kg)^0.425^

Heart rate (HR), systolic blood pressure (SBP) and diastolic blood pressure (DBP), were measured at the right brachial artery using a manual sphygmomanometer after the participants had rested for 10 minutes in a sitting position. Pulse pressure (PP) was calculated as the difference between SBP and DBP.

### Blood sampling for biochemical data

Blood samples were obtained from an antecubital vein for determination of hematocrit, hemoglobin, glucose, high density lipoprotein (HDL), and triglycerides. Samples were analyzed using the Hitachi 7170 Automatic Analyzer (Hitachi Corp. Hitachinaka Ibaraki, Japan) in accordance with the standard requirements dictated by the Clinical Laboratory Standards Institute (CLSI) guidelines (Specimen Choice, Collection, and Handling; Approved Guideline H18-A3).

### Determination of Nt-ProBNP levels

Serum biomarker N-terminal pro-brain natriuretic peptide (Nt-proBNP) was determined by an electrochemiluminescence immunoassay “ECLIA” assay (Roche Diagnostics GmbH, D-68298 Mannheim). After ensuring individualized patient samples, calibrators and controls were placed at ambient temperature (20–25°C) and were measured within 2 hours because of possible evaporation effects.

### Carotid artery imaging and cardiac geometries assessment

Transthoracic Doppler echocardiography was performed by using a commercialized ultrasound system (Vivid i, GE Vingmed) equipped with a 2- to 4-MHz transducer in all subjects. Parameters including LV internal diameter (either systolic or diastolic), wall thickness (septal or posterior wall thickness) and derived LV mass, and LV mass index were determined from M-mode measurements [10]. The definition of left ventricular hypertrophy (LVH) was defined by using gender-specific LV mass index (>115gm/m^2^ for men, and >95gm/m^2^ for women) data utilizing standardized formula [10].

The extra-cranial carotid arteries were assessed using a high-resolution ultrasound scanner (Acuson Aspen, Mountain View, CA) equipped with a 5-10-MHz linear-array transducer. The high-resolution B-mode ultrasonography evaluation protocol was performed at room temperature following 5 to 10 minutes of resting in the supine position [11]. Images were acquired, digitally recorded and analyzed by an experienced technician. The common carotid artery diameter (CCAD) was measured at the distal 1 cm of each common carotid artery, as the distance between the leading edges of the near- and far-wall intima at end-diastole by ECG gating [8]. Measurements were obtained in triplicate, and values from both right and left common carotids were averaged to give the summary CCAD value in each participant. In our lab, the reproducibility of inter-observer variability had been tested in a random 30 study subjects for carotid CCAD, LV septal wall thickness, and diameter (end-diastolic phase) which showed coefficient of variance (COV) of 5.6% [8], 7.4% and 6.8%, respectively.

### Statistical method

Baseline characteristics were compared between men and women using the Student’s t-test for normally distributed continuous variables and Chi-square test for discrete variables. Logistic regression was used for CCAD for identifying abnormal cardiac geometry as LVH, or significantly elevated Nt-ProBNP (> = 300pg/mL) [12] with odds ratio (OR) reported. Receiver operating characteristic curves were used to calculate the area under curve (AUROC) to identify LVH or abnormally elevated Nt-ProBNP (> = 300pg/mL), with optimal cut-off set at largest summation of sensitivity and specificity. Generalized estimating equations (GEE) were used to account for repeated measurements within individuals. To identify significant predictors of CCAD, univariable analyses were performed using both linear regression and GEE methods. Only clinical covariates which were significantly associated with CCAD in univariable analyses were included in multivariable analyses. Due to collinearity among the various blood pressure components (SBP, DBP and PP), these variables were included separately in multivariable models. We constructed sex-specific graphs depicting the association of CCAD with age and various blood pressure components (SBP, DBP and PP), as well as their sex-interactions, using longitudinal GEE models.

We further explored the combined effects of age, hypertension, and obesity on CCAD in sex-stratified analyses, where age groups (younger versus older) were defined using sex-specific median ages (47 years in women and 46 years in men), overweight was defined as BMI above 23kg/m [2, 13], and using younger non-hypertensive healthy weight women or men as the referent group.

All the analyses were performed by using SPSS 15 (SPSS Inc., Chicago, IL) and STATA software, version 8.2 (Stata Corp., College Station, Texas). All *p* values were two-sided with a value less than 0.05 considered statistically significant.

## Results

### Baseline clinical characteristics

A total of 998 participants (mean age: 47 ± 8.9 years, 66.4% men) were studied at baseline visit (Table 1). Despite similar mean age in men (46.8±9.0 years) and women (47.3±8.8 years) at baseline, men had higher SBP, DBP, BMI, fasting glucose, and triglyceride than women (all *p* < 0.001). Conversely, women had higher HDL levels than men (*p* < 0.001). Compared to men, women had significantly higher Nt-ProBNP levels (p<0.001). There were no significant sex differences in total cholesterol, pulse pressure or prevalence of hypertension or diabetes. In terms of lifestyle factors, men were more likely to be smokers than women, whereas both sexes were similarly likely to have regular exercise.

**Table 1:** Characteristics of participants at baseline (first visit, *n* = 998).

	Male (*n* = 663)	Female (*n* = 335)	t-test
Parameter	M±SD	M±SD	*P* value
Age (yr)	46.8±9.0	47.3±8.8	0.434
Anthropometric parameters			
Height (cm)	169.2±5.9	157.6±5.5	<0.001
Weight (kg)	70.5±9.5	56.3±8.5	<0.001
BMI (kg/m^2^)	24.6±2.9	22.6±3.0	<0.001
BP components			
SBP (mm Hg)	122.2±15.3	117.2±17.5	<0.001
DBP (mm Hg)	77.3±10.0	73.2±10.5	<0.001
Pulse pressure	44.9±9.9	44.0±11.0	0.202
Biochemical data			
Fasting Glucose (mg/dL)	97.9±20.1	94.2±18.1	0.005
Triglyceride (mg/dL)	140.8±99.1	93.5±61.1	<0.001
HDL (mg/dL)	49.5±12.0	62.8±15.3	<0.001
Common Carotid Artery Diameter (mm)			
CCAD (mm)	7.18±0.64	6.65±0.66	<0.001
Biomarker			
Nt-ProBNP (ng/L)	34.93±54.9	51.8±33.8	<0.001
Medical Histories, %			
Hypertension, %	62 (9.4%)	37 (11%)	0.394
Diabetes, %	17 (3%)	5 (2%)	0.269
Life Styles, %			
Current Smokers, %	134 (20%)	24 (7%)	<0.001
Regular Exercise, %	71 (11%)	27 (8%)	0.224

*Note*: All the variables were presented as mean±standard deviation.

*Abbreviations*: BMI, body mass index; SBP, systolic blood pressure; DBP, diastolic; HDL, high-density lipoprotein cholesterol; CCAD, common carotid artery diameter

Mean CCAD at baseline visit was larger in men (7.16±0.63 mm) than women (6.62±0.64 mm). This difference persisted after adjusting for differences in blood pressure (SBP, DBP and PP entered sequentially), BMI and cardiovascular risk factors (adjusted *p* < 0.001).

### The association among CCAD, cardiac geometries and Nt-Pro-BNP level at baseline and longitudinal data

We observed linear association between greater CCAD and larger LV mass index after accounting for confounding variables including age, gender, blood pressures and medical histories, either in cross-sectional data (Coef: 6.72, 95% CI: 4.38 to 9.06, p<0.001) or at longitudinal follow up (Coef: 6.69, 95% CI: 4.23 to 9.15, p<0.001) (Fig 2A).

In addition, there was a linear relationship between larger CCAD and higher LV mass index (β-coef: 6.72, p<0.05), higher prevalence for LVH (OR: 1.47, 95% CI: 1.06 to 2.07, p<0.05), and higher serum Nt-ProBNP level (β-coef: 5.35, OR: 4.22, 95% CI: 1.42 to 12.6 for Nt-ProBNP> = 300pg/mL, p<0.05) at baseline (Fig 2B), which remained significant after accounting for confounders. The AUROC and optimal cut-off for CCAD in identifying baseline LVH and abnormally high Nt-ProBNP was 7.25 and 7.95mm (AUROC: 0.65 & 0.79), respectively. Additionally, larger CCAD at longitudinal follow up was also associated with higher Nt-ProBNP level (Coef: 6.46, p = 0.019, Fig 2B) after adjusting for confounders (Coef: 6.46, p = 0.019).

### Cross-sectional association of CCAD with clinical characteristics

At baseline visit, clinical covariates that were cross-sectionally associated with larger CCAD included older age, larger BMI, higher blood pressure components (SBP, DBP, PP), higher blood glucose, higher triglyceride levels, lower HDL, a history of hypertension or diabetes, current smoking and regular exercise (Table 2). After multivariable adjustment, older age, larger BMI, elevated blood pressure components, current smokers remained independently associated with larger CCAD at baseline.

**Table 2:** Cross-sectional association of CCAD with clinical characteristics at baseline (visit 1, *n* = 998).

	At Baseline							
	Univariable Model		MV Models (SBP)		MV Models (DBP)		MV Models (PP)	
Predictor Covariates	*B*	*P*	*B*	*P*	*B*	*P*	*B*	*P*
Age (yr)	0.21	<0.001	0.18	<0.001	0.19	<0.001	0.19	<0.001
Male Gender	0.45	<0.001	0.43	<0.001	0.42	<0.001	0.46	<0.001
Anthropometrics								
BMI (kg/m^2^)	0.24	<0.001	0.09	0.004	0.09	0.005	0.11	0.001
BP Components								
SBP (mm Hg)	0.23	<0.001	0.11	<0.001	—	—	—	—
DBP (mm Hg)	0.20	<0.001	—	—	0.1	<0.001	—	—
Pulse pressure (mm Hg)	0.16	<0.001	—	—	—	—	0.06	0.001
Biochemical data								
Fasting Glucose (mg/dL)	0.16	<0.001	0.05	0.076	0.06	0.05	0.06	0.057
Triglyceride (mg/dL)	0.12	<0.001	-0.04	0.227	-0.03	0.262	-0.04	0.233
HDL (mg/dL)	-0.15	<0.001	0.01	0.795	0.01	0.816	0.01	0.856
Medical Histories								
Hypertension	0.48	<0.001	0.05	0.547	0.06	0.467	0.08	0.285
Diabetes	0.53	<0.001	0.14	0.375	0.13	0.421	0.17	0.290
Life Styles								
Current Smokers	0.22	<0.001	0.15	0.008	0.15	0.01	0.15	0.01
Regular Exercise	0.17	0.025	-0.06	0.421	-0.05	0.444	-0.07	0.329

*Note*: Regression coefficients represent the change in mean difference in CCAD (in mm) per 1-SD difference in each continuous predictor variable; In multivariable analysis, DBP and LDL were excluded due to the high correlation with SBP and Cholesterol, respectively.

MV: multivariable.

### Longitudinal association of CCAD with clinical characteristics during repeated visits (GEE models)

Accounting for repeated visits using GEE models, clinical covariates that were associated with increasing CCAD included older age, larger BMI, higher blood pressure components (SBP, DBP, PP), higher blood glucose, higher triglyceride levels, lower HDL, and a history of hypertension or diabetes (Table 3). After multivariable adjustment, older age, larger BMI, and elevated blood pressure components remained independently associated with increasing CCAD at repeated visits. For models including SBP, each 1SD increase in age, SBP, and BMI was associated with an increase of CCAD by 0.12, 0.05, and 0.07 mm respectively (Table 3). For models including DBP, CCAD increased by 0.13, 0.02, and 0.07 mm for each 1SD increase in age, DBP, and BMI respectively; while for models including PP, CCAD increased by 0.13, 0.03 & 0.08 mm for each 1SD increase in age, PP, and BMI respectively. For all models, men had larger CCAD than women.

**Table 3:** Longitudinal association of CCAD with clinical characteristics during repeated visits (*n* = 2,914).

	Longitudinal GEE (All Cohort)							
	Univariable Model		MV Models (SBP)		MV Models (DBP)		MV Models (PP)	
Predictor variable	*B*	*P*	*B*	*P*	*B*	*P*	*B*	*P*
Age (yr)	0.11	<0.001	0.12	<0.001	0.13	<0.001	0.13	<0.001
Male Gender	0.52	<0.001	0.46	<0.001	0.46	<0.001	0.47	<0.001
Anthropometrics								
BMI (kg/m^2^)	0.14	<0.001	0.07	0.001	0.07	<0.001	0.08	<0.001
BP Components								
SBP (mm Hg)	0.04	<0.001	0.05	<0.001	—	—	—	—
DBP (mm Hg)	0.02	0.003	—	—	0.02	0.007	—	—
Pulse pressure (mm Hg)	0.02	<0.001	—	—	—	—	0.03	<0.001
Biochemical Data								
Fasting Glucose (mg/dL)	0.02	0.016	0.01	0.65	0.01	0.504	0.01	0.507
Triglyceride (mg/dL)	0.018	0.046	-0.02	0.193	-0.02	0.198	-0.02	0.243
HDL (mg/dL)	-0.03	0.005	-0.01	0.587	-0.03	0.004	-0.01	0.348
Medical Histories								
Hypertension	0.05	0.035	0.05	0.063	0.05	0.069	0.05	0.1
Diabetes	0.1	0.038	0.1	0.089	0.11	0.054	0.1	0.083
Life Styles								
Current Smokers	0.034	0.152	0.01	0.768	0.01	0.774	0.01	0.843
Regular Exercise	0.028	0.165	0.02	0.468	0.02	0.499	0.01	0.579

### Sex-stratified analyses of the effects of age, hypertension and obesity on CCAD remodeling at baseline

**Fig 3:**
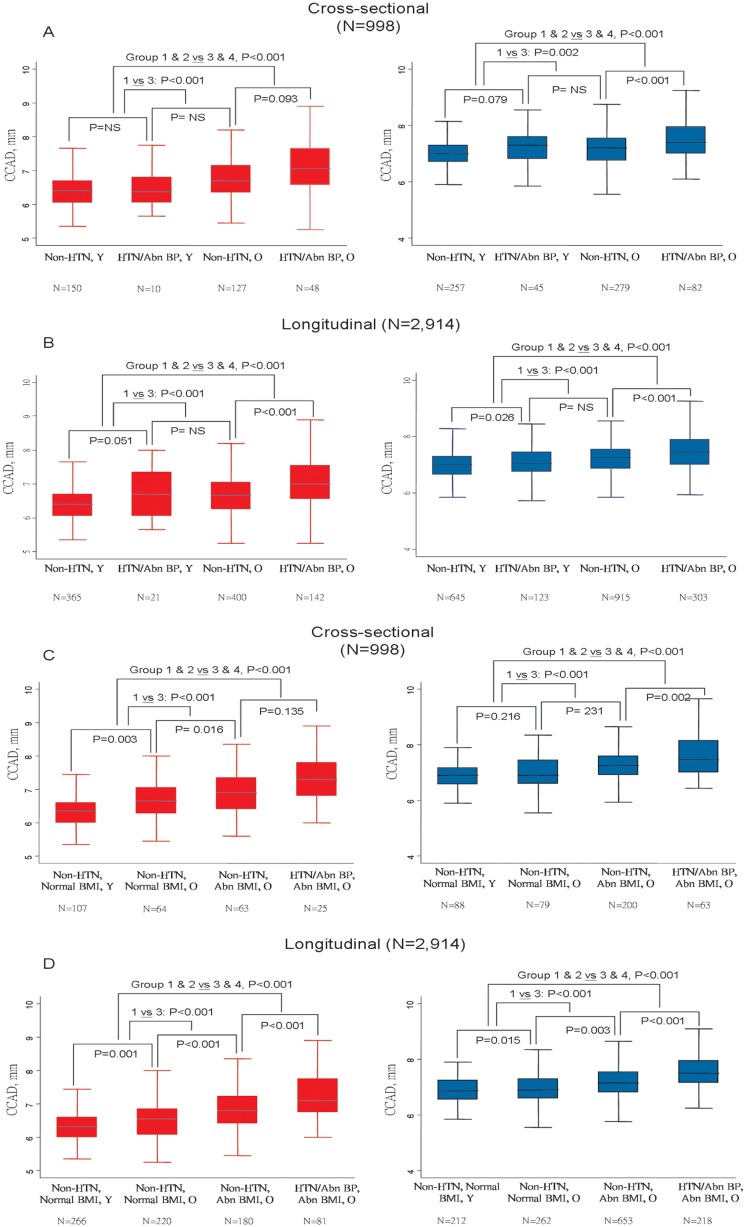
Sex-specific comparisons and illustrations of CCAD in categorized abnormal blood pressure (defined by SBP>140 or DBP>90mmHg), hypertensive status (A & B), body size in terms of BMI (C & D) and different age groups from both baseline visit and longitudinal follow up data. BMI: body mass index, Non-H: non-hypertensives, H: hypertensives. Y: young group, O: old group, based on sex-specific median ages. ★ p<0.05 compared to Non-H group (A-C) or <40 age categories (D).

The association between CCAD, age (categorized as young: Y or old: O based on sex-specific median ages), blood pressure, hypertensive status and obesity status in terms of BMI for both baseline (n = 998) and longitudinal data (n = 2,914) were shown in Fig 3. Subjects with abnormal blood pressure (either SBP>140 or DBP>90mmHg) or those with known HTN history were categorized as HTN/Abn BP group, while subjects with BMI larger than 23kg/m^2^ were categorized as Abn BMI group. We showed that both men and women had similar trend of having greater CCAD for subjects with increasing age (>47 years in women and >46 years in men) or those with abnormal blood pressure or hypertensive status (Fig 3A and 3B, all p for trend <0.001). In addition, similar trends (Fig 3C and 3D, all p for trend <0.001) were observed for those subjects with increasing age or excessive obesity in terms of larger BMI (Fig 3C and 3D, all p for trend <0.001) in both genders.

### Sex modification of the association between age and blood pressure on CCAD remodeling

**Fig 4:**
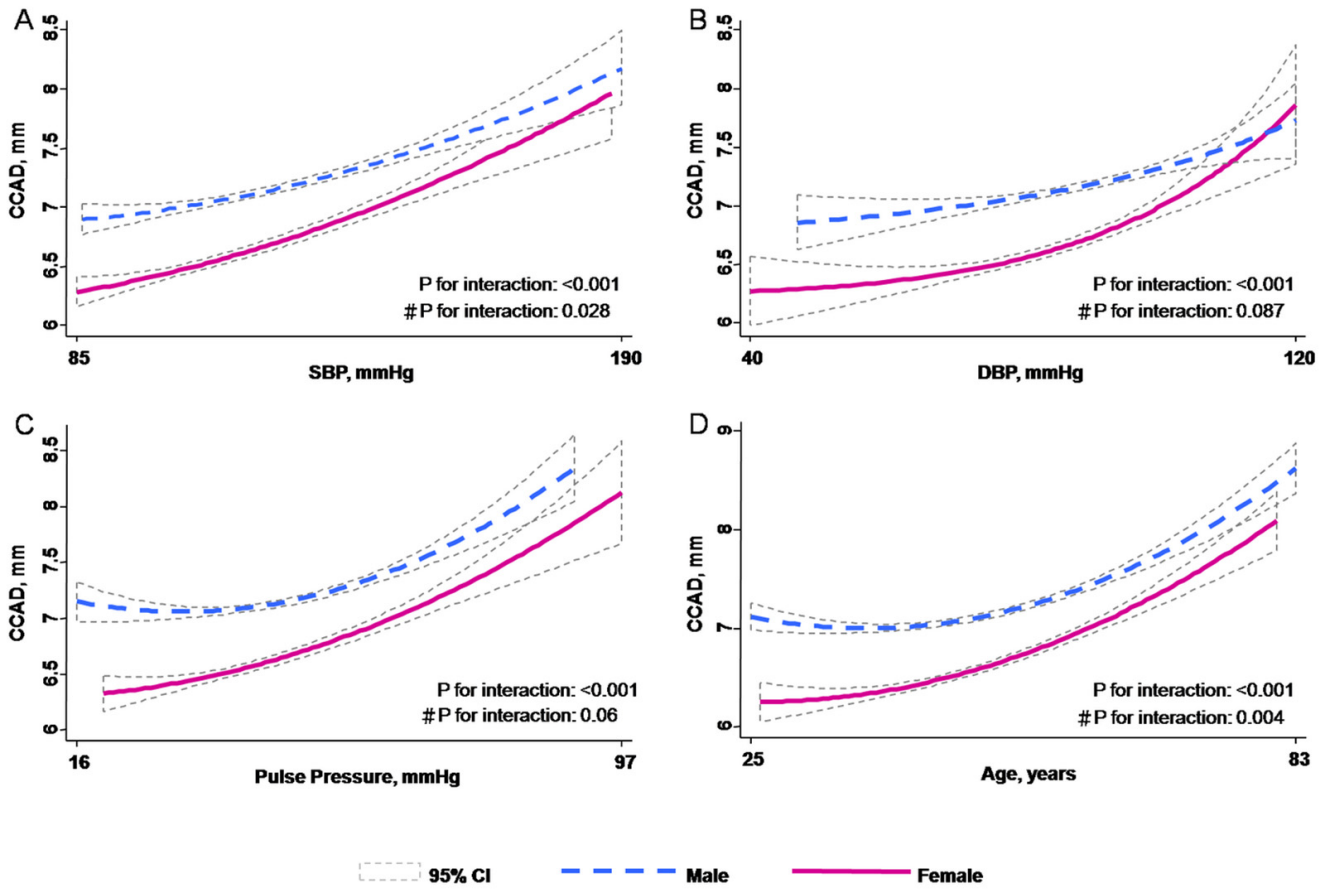
Sex-specific predictive curves for common carotid artery diameter (CCAD) with (A) SBP, (B) DBP, (C) PP, (D) Age in the longitudinal GEE model. P for interaction between CCAD and genders were illustrated with or without adjustment. # after adjusting clinical covariates.

Sex was a significant effect modifier in the relationship between age and CCAD, as well as between blood pressure and CCAD. These effects are illustrated in Fig 4. The slope of the increase in CCAD with increasing blood pressure was steeper in women than men, for all blood pressure components in unadjusted analyses (Fig 4A–4C, all p for interaction: <0.001), and for SBP following multivariable adjustment (β coef: 0.05 & 0.01 for women and men, respectively, p for interaction: <0.05). Similarly, the slope of the increase in CCAD with increasing age was steeper in women than men, before and after multivariable adjustment (Fig 4D) (β coef: 0.17 & 0.1 for women and men, respectively, p for interaction: <0.05). There was no significant interaction between sex and BMI on CCAD remodeling.

## Discussion

These longitudinal data in a large cohort of asymptomatic Asian adult cohort undergoing serial health screening show that larger CCAD was consistently associated with greater LV mass index and higher serum Nt-ProBNP level, both are clinical structural or functional surrogates for unfavorable cardiac remodeling, either at baseline or longitudinal data. Furthermore, we observed that age, body size and blood pressure are all independent determinants of carotid artery remodeling. Prominent sex differences were observed, where, despite smaller CCAD at baseline, women demonstrated greater rates of carotid artery dilatation with increasing age and blood pressure than men. These sex differences in arterial remodeling may carry implications for the development of sex-specific cardiovascular disease, and deserve further study.

Remodeling or dilatation of large elastic arteries (such as the carotid arteries) has been shown to be related to increased mechanical stress from high pulsatile loads, leading to breakdown of elastic fibers within the arterial walls [8,14,15]. Several cardiovascular risk factors including increasing age, higher blood pressures, larger body size, and unfavorable biochemical profiles (e.g. blood glucose, triglycerides and HDL cholesterol level) may all contribute to excessive proteoglycan/collage matrix deposition and smooth muscle proliferation, leading to a thickened intima-media layer and increased atherosclerotic plaque burden, which actually shared common features for heart failure development [4, 8, 16, 17]. Adaptive changes to preserve the arterial lumen diameter in response to “inward” arterial wall thickening may also explain the resultant “outward” arterial dilatation [6, 8, 12, 18]. The effects of arterial remodeling include reduced elastance [19] and coupled ventricular-arterial stiffening [5, 20, 21, 22, 23].

We previously showed that carotid artery remodeling was a useful clinical marker of early stage myocardial dysfunction and elevated plasma BNP level, even when left ventricular ejection fraction was normal [8]. Further, LV mass index as a common cardiac remodeling parameter had recently been shown to be a strong predictor for subsequent development of heart failure [24, 25]. While both CCAD and biomarkers of heart failure such BNP may increase in parallel as surrogates of cardiac functional disturbances, data regarding the linear relationship among CCAD, LV mass index and serum Nt-ProBNP level remains largely unknown, especially in a relatively large, asymptomatic cohort. It is noteworthy that female sex, aging, hypertension, and obesity—all key risk factors of carotid arterial remodeling in our current study—are also the known main risk factors for heart failure with preserved ejection fraction, which was further supported by the consistent linear relationship between larger CCAD and serum Nt-ProBNP level in our current work [26–32]. In aggregate, our findings are consistent with a concept of combined arterial-ventricular remodeling which is accentuated in elderly hypertensive or obese women, and may contribute to their predisposition to heart failure with preserved ejection fraction [33–34].

### Study Limitations

We are unable to investigate the association of carotid artery remodeling with future heart failure in this study due to our lack of outcome data. Nonetheless, our large cohort and availability of repeated measurements provided novel longitudinal data and statistical power to examine the relationships of interest. Further, effects of anti-hypertensive may have some beneficial effects on vascular remodeling process. Even though, our results were robust and consistent from both cross-sectional and longitudinal analyses in that higher blood pressure remains as independent factor in such remodeling process, while any use of these therapeutic agents were not statistically significant in multivariable models. Finally, our results apply to an asymptomatic Asian cohort, and further study is needed to ascertain the generalizability of these findings to other populations.

## Conclusion

We demonstrated in our current work that greater CCAD was associated with larger LV mass index and higher Nt-ProBNP level in asymptomatic population. Therefore, CCAD may serve as an alternative clinical marker for unfavorable cardiac remodeling process even at pre-clinical stage. In addition, our longitudinal data also demonstrate that the major determinants of carotid artery remodeling in asymptomatic adults are age, body size and blood pressure. Despite smaller CCAD at baseline, women have greater rates of carotid artery dilatation with increasing age and blood pressure than men. These sex differences in arterial remodeling may carry implications for the development of sex-specific cardiovascular disease, and deserve further study.
